# Intérêt du sulfate de magnésium pour la stabilitée hémodynamique en cœliochirurgie: étude prospective contrôlée randomisée

**DOI:** 10.11604/pamj.2024.47.215.41212

**Published:** 2024-04-29

**Authors:** Imen Zouche, Salma Ketata, Mariem Bousarsar, Faiza Grati, Rahma Derbel, Nizar Kardoun, Sami Fendri, Hichem Cheikhrouhou

**Affiliations:** 1 Service d’Anesthésie Réanimation Chirurgicale, Centre Hospitalier Universitaire Habib Bourguiba Sfax, Sfax, Tunisie,; 2 Service de Chirurgie Viscérale, Centre Hospitalier Universitaire Habib Bourguiba Sfax, Sfax, Tunisie

**Keywords:** Sulfate de magnésium, cholésystectomie, cœliochirurgie, pneumopéritoine, stabilité hémodynamique peropératoire, Magnesium sulphate, cholecystectomy, laparoscopic surgery, pneumoperitoneum, intraoperative haemodynamic stability

## Abstract

**Introduction:**

au cours de la chirurgie laparoscopique, l'insufflation du dioxyde de carbone pour la création de pneumopéritoine augmente la pression artérielle, la fréquence cardiaque et la résistance vasculaire systémique. Le but de notre étude était d´étudier l'efficacité de sulfate de magnésium dans la prévention des réactions hémodynamiques indésirables associées au pneumopéritoine chez les patients subissant une cholécystectomie laparoscopique.

**Méthodes:**

nous avons mené une étude clinique prospective contrôlée randomisée en double aveugle incluant des patients proposés pour cholécystectomie laparoscopique et répartis en deux groupes équivalents: le groupe Mg^2+^ ayant reçu en intraveineux lent 50 mg/kg de sulfate de magnésium avant l'insufflation de pneumopéritoine et le groupe S ayant reçu le même volume de 0,9 % de solution saline. Notre critère de jugement principal était les variations de la pression artérielle systolique (PAS) peropératoires relatives au pneumopéritoine notamment à une minute après l'insufflation. Les critères de jugement secondaires étaient le retentissement hémodynamique du pneumopéritoine en termes de PAS, pression artérielle diastolique (PAD), pression artérielle moyenne (PAM), et fréquence cardiaque (FC) de deux minutes après l´insufflation jusqu´à l´extubation et en post opératoire ainsi que la présence d´éventuels effets indésirables liés à l´administration du sulfate de magnésium.

**Résultats:**

nous avons inclus 70 patients répartis en deux groupes de 35. La PAS était significativement supérieure chez le groupe S à l'insufflation (T0), 3 min, 4 min et 5 min après l'insufflation, et à 60 min post opératoire. La FC était significativement plus élevée chez les patients du groupe S par rapport au groupe Mg^2+^ à 7 min et 8 min après l'insufflation. Aucune différence significative des mesures de la PAD et de la PAM n'a été observée entre les deux groupes. Aucun effet indésirable lié à l'administration de magnésium n´a été noté.

**Conclusion:**

le sulfate de magnésium administré avant l'insufflation du pneumopéritoine a assuré une meilleure stabilité hémodynamique peropératoire au cours de la coeliochirurgie.

## Abstract (en)

**Introduction:**

during laparoscopic surgery, carbon dioxide (CO_2_) insufflation to create pneumoperitoneum increases blood pressure, heart rate and systemic vascular resistance. The purpose of our study was to investigate the efficacy of magnesium sulfate in preventing adverse hemodynamic reactions associated with pneumoperitoneum in patients undergoing laparoscopic cholecystectomy.

**Methods:**

we conducted a prospective, randomized, double-blind, controlled clinical study of patients scheduled for laparoscopic cholecystectomy and divided into two equal groups: the Mg^2+^ group received slow intravenous magnesium sulfate 50 mg/kg injection prior to pneumoperitoneum insufflation while the S group received the same volume of 0.9 % saline. Our primary endpoint was intraoperative changes in systolic blood pressure (SBP) related to pneumoperitoneum, in particular at 1 minute after insufflation. The secondary endpoints were the haemodynamic effects of pneumoperitoneum in terms of systolic blood pressure (SP), diastolic blood pressure (DP), mean arterial pressure (MAP) and heart rate (HR) from 2 minutes after insufflation to extubation and postoperatively, and the presence of possible adverse reactions related to the administration of magnesium sulphate.

**Results:**

we included 70 patients divided into two groups of 35. SP was significantly higher in the S group at insufflation (T0), 3 min, 4 min and 5 min post-operative, and at 60 min after surgery. HR was significantly higher in patients in the S group compared to the Mg^2+^ group at 7 min and 8 min after insufflation. No significant differences in DP and MAP measurements were observed between the 2 groups. No adverse reactions related to magnesium administration were reported.

**Conclusion:**

magnesium sulfate administered prior to pneumoperitoneum insufflation provided improved intraoperative hemodynamic stability during laparoscopic surgery.

## Introduction

La cholécystectomie par voie laparoscopique est le gold standard dans la chirurgie biliaire depuis 1987 [1]. Les interventions chirurgicales par voie laparoscopique visent à obtenir un résultat thérapeutique satisfaisant tout en minimisant le stress traumatique et métabolique de l'intervention diminuant ainsi le risque de complications post opératoires. Au cours de la cholécystectomie laparoscopique, le dioxyde de carbone est couramment utilisé pour créer le pneumopéritoine [2,3]. Le dioxyde de carbone (CO_2_) et le pneumopéritoine causent des effets hémodynamiques indésirables [4] notamment la baisse du débit cardiaque qui est secondaire à la diminution du retour veineux engendré par l´augmentation de la pression intra abdominale et l´élévation abrupte de la pression artérielle et de la résistance vasculaire systémique suite à la libération accrue de catécholamine et de vasopressine [5,6]. Ce qui peut être néfastes pour les patients dont la fonction cardiaque est compromise [7]. Le magnésium a la capacité de bloquer la libération de catécholamines de la glande surrénale et des terminaisons du nerf adrénergique [8]. Il peut aussi produire une vasodilatation en agissant directement sur les vaisseaux sanguins [9] et il peut également atténuer la sécrétion de vasopressine stimulée par la vasoconstriction [10]. Le but de notre étude était d´évaluer l´effet du sulfate de magnésium administré par voie intraveineuse sur la réponse hémodynamique secondaire à l'insufflation du pneumopéritoine chez les patients subissant une cholécystectomie laparoscopique programmée.

## Méthodes

**Conception et cadre de l´étude:** il s'agit d'une étude clinique randomisée, prospective, contrôlée en double aveugle, portant sur une cohorte de patients proposés pour une cholécystectomie coelioscopique programmée.

**Population étudiée:** nous avons inclus des patients âgés entre 20 et 65 ans classés selon le score de *l'American Society of Anesthesiologist (ASA)*, ASA I et ASA II proposés pour une cholécystectomie laparoscopique programmée suite à une lithiase vésiculaire simple. Les critères de non inclusion étaient: les patients ayant une intubation prévue difficile, une allergie connue au sulfate de magnésium, une insuffisance rénale, une insuffisance respiratoire, une maladie neuromusculaire, les patients traités par des inhibiteurs calciques et l´obésité morbide (index de masse corporelle >40 kg/m^2^). Nous avons exclu la conversion en laparotomie et le non-respect du protocole anesthésique choisi.

**Calcul de la taille de l´échantillon:** la détermination du nombre de patients nécessaires a été réalisée à partir des résultats d´une pré-enquête effectuée sur 60 patients. Le calcul du nombre des sujets nécessaire a été effectué pour avoir un écart de PAS minimum de 20mmHg entre les deux groupes. Sur la base de ces estimations, nous avons calculé une taille d´échantillon permettant une erreur de type alpha de 0.05 avec une puissance β de 95 %. Il a été estimé nécessaire que chacun des deux groupes soit formé par un minimum de 25 individus. Nous avons alors désigné 35 patients par groupe pour avoir le nombre suffisant après d´éventuelles exclusions.

**Randomisation:** la randomisation a été effectuée à l´entrée du bloc opératoire après vérification des critères d´inclusion et de non inclusion. Elle a été basée sur des codes générés par ordinateur conservés dans des enveloppes opaques numérotées séquentiellement selon un ratio de 1: 1. Le tirage au sort a été effectué par un médecin anesthésiste autre que celui qui a assuré l´anesthésie et le suivi post opératoire. Les patients ont été répartis au hasard en deux groupes:

Groupe Mg^2+^: patients recevant avant l'induction anesthésique une perfusion de 50 mg/kg de sulfate de magnésium dans 100cc de sérum physiologique sur 15 min.

Groupe S: patients recevant avant l'induction anesthésique une perfusion de 100cc de sérum physiologique sur 15 min.

### Intervention

L'étude a été menée par 2 médecins anesthésiste réanimateur. L'un a été chargé par la préparation des seringues selon la randomisation dans les règles de l´asepsie rigoureuse, tandis que l´autre a été chargé du suivi per et post-opératoire. Les mêmes procédures anesthésiques et chirurgicales ont été appliquées pour tous les patients. A la salle d'opération, le monitorage a compris la surveillance de la pression artérielle non invasive, la fréquence cardiaque, La fréquence respiratoire, La saturation pulsée en oxygène et la Pet CO_2_. Une voie veineuse périphérique de calibre 18 G a été mise en place permettant la perfusion de la solution selon la randomisation (soit 100cc de sérum physiologique soit 50mg/kg de sulfate de magnésium dans 100cc de sérum physiologique) sur 15 min. Tous les patients ont bénéficié d'une compensation hydrique systématique du jeune préopératoire. Après dénitrogénation au masque à 100 % d´oxygène durant 3 minutes, une anesthésie générale a été induite avec 3μg/kg de fentanyl, 2-3 mg/kg de propofol et 0,15mg/kg de cisatracrium (Nimbex®) suivie d´une ventilation au masque pendant 3 minutes puis intubation orotrachéale par une sonde adaptée à la taille et du poids du patient. L'entretien a été assuré par des réinjections de fentanyl 50 à 100 µg et de cisatracrium 0.03 mg/kg toutes les 40 minutes avec le sévoflurane 2 % à 3 % dans 50 % d'oxygène / air. Une ventilation à assistée contrôlée a été adoptée, le volume courant et les fréquences respiratoires ont été ajustés pour maintenir le taux de CO_2_ expiré entre 35 et 40 mm Hg. Une sonde nasogastrique a été insérée. Une minute après l´intubation, les trocarts ont été introduits et la cavité péritonéale a été insufflée de dioxyde de Carbone. Une pression intra-abdominale à 12 mmHg a été maintenue durant toute la période chirurgicale.

Les pressions artérielles systolique (PAS), diastolique (PAD) et moyenne (PAM), la fréquence cardiaque (FC) ont été notés avant l'induction puis chaque min durant 10 min puis toutes les 5 min jusqu´à l´extubation puis à la salle de surveillance post interventionnelle (SSPI) toutes les 15 minutes pendant 2 heures. Les variations hémodynamiques peropératoire ont été identifiées. Les épisodes d´hypotension définie par une PAS < 25 % de la PAS de base ont été traités par des boli de 3 mg d'éphédrine et les épisodes de bradycardies définies par un pouls inférieur ou égal à 50 battements/minute ont étés traitées par des boli d´atropine 20μg /kg. Les épisodes d´HTA définies par une PAS supérieure ou égale à 140 mmHg ont été traités par des boli de 1ml de nicardipine (Loxen®) intraveineux et les épisodes de tachycardie définies par un pouls >90 battements/min ou pouls qui est > 150 % du pouls initial ont été traitées par un remplissage adéquat de 30 ml/kg de cristalloïdes, et dans les cas où la tachycardie persiste, on a recours au bétabloquant (Sectral®). Trente minutes avant l´extubation, 1 g de paracétamol (Perfalgan®), avec 20 mg de nefopam (Acupan®) ont été administrés en intraveineux sur 30 mm. A la fin de l´acte, les patients ont été extubés sur la table opératoire puis transférés à la SSPI pendant deux heures puis au service de chirurgie.

**Les données recueillies:** en préopératoire, nous avons recueilli l´âge, la classe ASA, les antécédents médicaux et chirurgicaux. En peropératoire, nous avons recueilli la durée de l´acte calculée à partir du moment de l'incision ombilicale pour l'insertion du trocart jusqu'à la mise en place du dernier point de suture et la durée de l´anesthésie calculée depuis l´induction jusqu'à l´extubation. La fréquence cardiaque (FC), saturation pulsée en O_2_(SPO_2_), la pression artérielle systolique (PAS), la pression artérielle diastolique (PAD), pression artérielle moyenne (PAM) ont été noté avant l´induction, chaque min durant 10min puis toutes les 5 min jusqu´à l´extubation et en post opératoire toutes les 15 minutes pendant les 2 premières heures.

**Critères de jugement:** notre critère de jugement principal était les variations de la pression artérielle systolique (PAS) peropératoires relatives au pneumopéritoine entre les deux groupes notamment à 1 minute après l´insufflation. Les critères de jugement secondaires étaient le retentissement hémodynamique du pneumopéritoine en termes de PAS, PAD, PAM, et FC à 2 min après l´insufflation jusqu´à l'extubation et en post opératoire ainsi que la présence d'éventuels effets indésirables liés à l´administration du sulfate de magnésium.

### Etude statistique

La saisie et l´analyse des données ont été réalisées avec SPSS version 25.0. L'étude descriptive des variables qualitatives a été exprimée en pourcentages (%) et en effectifs de chaque modalité. Pour les variable quantitatives, les tests de normalité de Shapiro-Wilk et Kolmogorov Smirnov ont été réalisés. Les variables quantitatives ayant une distribution normale étaient exprimées en moyenne ± déviation standard (ou écart type), et celles ayant une distribution non normale étaient décrites en médiane avec les extrêmes. Pour l'étude analytique, la comparaison des variables qualitatives sur séries indépendantes a été effectuée par le test de Chi-deux de Pearson et le test exact bilatéral de « Fisher » en cas d´effectif inférieur à cinq. Pour les variables quantitatives normales, la comparaison de deux moyennes a été effectuée par le test de t-Student et par le test non paramétrique de Mann-Whitney quand la série n´obéit pas à la loi normale. Dans toutes les comparaisons, la valeur de p = 0,05 a été considérée comme statistiquement significative.

**Considération éthique:** cet essai clinique a été réalisé après accord du comité de protection des personnes sud C.P.P.SUD sous l´égide des ministères de la santé et de la justice de la république tunisienne (référence CPP SUD N° 25/2019) et après consentement éclairé et écrit des patients inclus.

## Résultats

### Caractéristiques générales de la population étudiée

**Figure 1:**
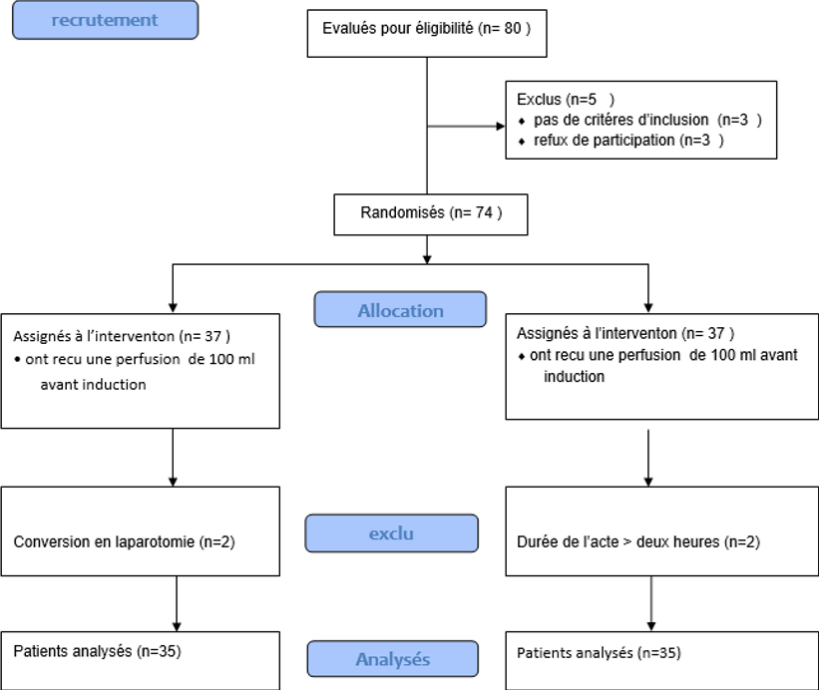
diagramme de flux

Nous avons inclus 74 patients dont quatre patients ont été exclus pour conversion en laparotomie. Soixante-dix (70) patients ont été analysés et répartis en deux groupes de 35 (Figure 1). La moyenne d´âge de notre échantillon était égale à 43,2±10,66 ans avec un sexe ratio de 0,62. Aucune différence significative des paramètres démographiques et comorbidités n'a été observée entre les deux groupes de notre étude (Tableau 1). La durée de l´acte opératoire a été comparable entre les deux groupes (Tableau 1). La pression d´insufflation n'a pas dépassé 12 mmHg dans les deux groupes de l´étude sans différence significative entre les deux groupes.

**Tableau 1:** comparaison des paramètres pré et peropératoires entre les deux groupes

Paramètres démographiques	Groupe Mg2+ (n=35)	Groupe S (n=35)	P
**Age (ans)±ET**	41,5 ± 10,69	44,9 ± 10,63	0,183**
**Sexe (H/F)**	13/22	14/21	0,806*
**IMC(Kg/m^2^) ± ET**	27,51 ± 3,85	27,11 ± 2,57	0,454**
**ASA (I/II)**	26/9	27/8	0,780*
**Durée moyenne de l'acte chirurgical (minutes) ± ET**	65 ± 24,66	63,27 ± 20,5	0,965**

Groupe Mg2+: Groupe magnésium; Groupe S: placebo; n: nombre; H: Homme, F: Femme; IMC: indice de masse corporelle; ASA: American society of Anesthesiologists, ET: Ecart Type Tests statistiques: **: Test de Student; *: Test de Chi-deux de Pearson

### Critère de jugement principal

**Figure 2:**
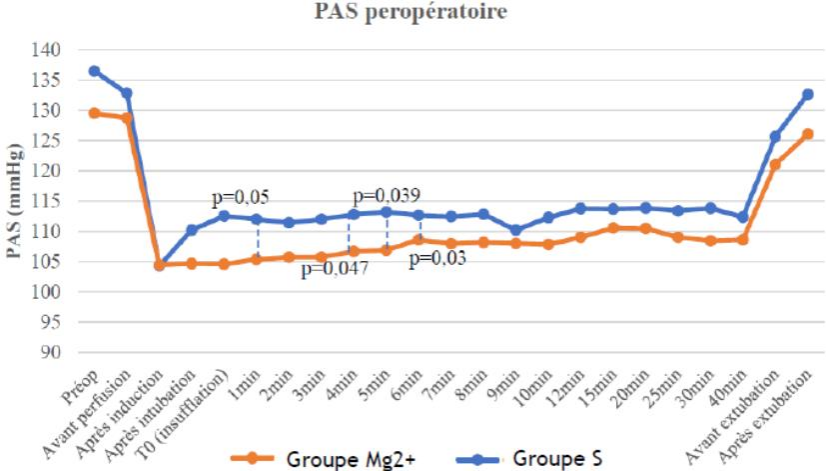
variation peropératoire des pressions artérielles systoliques (PAS) entre les deux groupes

En peropératoire la PAS était significativement supérieure dans le groupe S par rapport au groupe Mg^2+^ à l'insufflation (T0) (112,54±18,03 vs 104,62±18,03 mmHg, p=0.05), à 3 min (112±13,86 vs 105,74±13,86 mmHg, p= 0,047), à 4 min (1128±12,78 vs 106,71±12,78mmHg, p= 0,039), à 5 min (113,2±12,35 vs 106,91±12,35mmHg, p=0,03) après l´insufflation et après l´extubation (132,68±12,65 vs 126,08±12,65mmHg, p=0,028) (Figure 2). En postopératoire, La PAS était significativement plus élevée chez les patients du groupe S par rapport au groupe Mg^2+^ à 60 min post-opératoire (121±11,25 vs 113,85±11,25 mmHg, p=0,021).

### Critère de jugement secondaires

**Figure 3:**
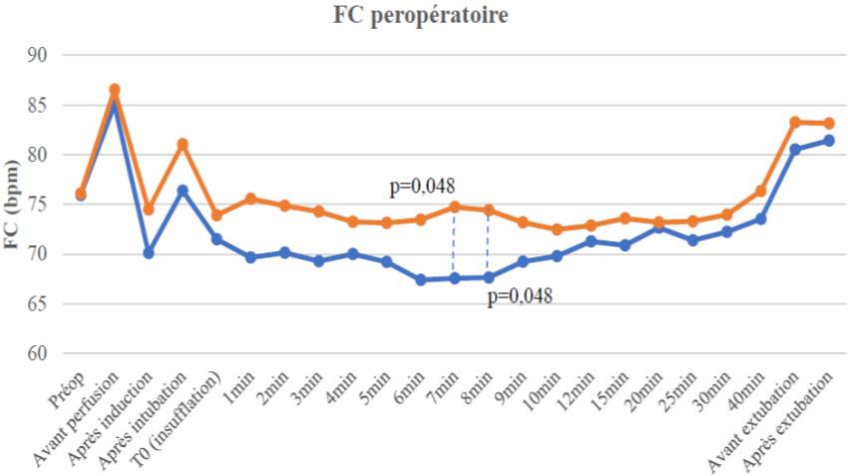
variation peropératoire des fréquences cardiaques (FC) entre les deux groupes

Aucune différence significative des mesures de la PAD et de la PAM n'a été observée entre les deux groupes en per et en postopératoire. La FC était significativement plus élevée chez les patients du groupe S par rapport au groupe Mg^2+^ à 7 min (74,71±17,43 vs 67,54±17,43 batt/min, p=0,048), à 8 min (74,4±16,05 vs 67,62±16,05 batt/min, p= 0,048) après l´insufflation du pneumopéritoine (Figure 3) et à 60 min postopératoire (84,17±11,76 vs77,97±11,76 batt/min, p=0,024). Aucune différence significative des mesures de la PetCO2 n'a été observée entre les deux groupes en peropératoire. Il n´y avait pas de différence significative entre les deux groupes en termes d´incidence des épisodes d´hypo ou d´hypertension artérielle et de tachy ou de bradycardie en per et en post opératoire (Tableau 2). L'hypercapnie était plus observée chez les patients du groupe Mg^2+^ par rapport au groupe S (31,4 % vs 22,9 %) sans aucune différence significative entre les deux groupes. Aucun effet indésirable lié à l'administration de magnésium n'a été noté.

**Tableau 2:** comparaison de l'incidence des troubles de rythme et de pressions artérielles entre les deux groupes

	Groupe Mg2+ (n=35)	Groupe S (n=35)	P
**Hypotension**	5 (14,3 %)	2 (5,7 %)	0,428ƚ
**Hypertension**	11 (31,4 %)	5 (14,3 %)	0,088ƚ
**Tachycardie**	11 (34.4 %)	13 (37.2 %)	0,403*
**Bradycardie**	4 (11,4 %)	7 (20 %)	0,324*

Groupe Mg2+: groupe magnesium, Groupe S: groupe sérum physiologique; n: effectif; Tachycardie= fréquence cardiaque > 90 bpm; Bradycardie = fréquence cardiaque < 50 bpm. Tests statistiques: *: Test de Chi-deux de Pearson, ƚ: Test de fischer

## Discussion

Nous avons mené une étude clinique prospective randomisée incluant 70 patients proposés pour cholécystectomie laparoscopique programmée et dont l´objectif était d´étudier l´effet de l´injection en intraveineux de sulfate de magnésium sur la réponse hémodynamique secondaire à l´insufflation du pneumopéritoine. Notre étude a montré que le sulfate de magnésium a assuré une meilleure stabilité hémodynamique avec une atténuation des variations de la PAS à l´insufflation (T0), 3 min, 4 min, 5 min après l´insufflation, et à 60 min postopératoire et de la FC à 7 min, à 8 min après l´insufflation du pneumopéritoine et à 60 min postopératoire sans effets indésirables notables.

L´insufflation intrapéritonéale de CO_2_ pendant la cœlioscopie entraîne une élévation de la pression intrapéritonéale responsable des retentissements cardiocirculatoires, respiratoires ainsi que des perturbations des perfusions d´organes suite à une compression de la veine cave inférieure [11] avec une réduction du retour veineux [12] et une diminution du débit cardiaque de 20 à 59 % qui est d´autant plus marquée que la volémie est basse [13]. Des réactions catécholaminergiques et humorales sont également impliquées dans les retentissements cardio-circulatoires du pneumopéritoine, avec une participation importante de la sécrétion de vasopressine en réponse à l´activation des volorécepteurs de l´oreillette droite entrainant une augmentation des résistances vasculaires systémiques responsables de l'augmentation de la pression [14,15]. Le magnésium est largement utilisé pour atténuer la réponse hémodynamique induite par l´intubation en bloquant la libération des catécholamines à la fois des médullosurrénales et des terminaisons nerveuses, de plus, en diminuant l´effet de la vasopressine [16,17] et en agissant directement sur les vaisseaux sanguins causant une vasodilatation [18-20].

L´étude de Showket Ahmad Dar *et al*. [21] a comparé 62 patients ayant subi une chirurgie abdominale laparoscopique qui ont été réparties en deux groupes au hasard: le groupe MG ayant reçu 50 mg /kg de sulfate de magnésium dilué dans une solution saline normale, 5 min après l´intubation et avant la création du pneumopéritoine, et le groupe témoin ayant reçu la même quantité de solution saline normale. Cette étude a montré que la pression artérielle systolique était significativement plus faible dans le groupe MG à 5, 10, 15, 20, 25, 30, 35, 40, 45, 50, 55, et 60 minutes après le pneumopéritoine par rapport au groupe Témoin. Aussi la pression artérielle diastolique était plus faible dans le groupe MG avant le pneumopéritoine et à 10, 15, 20,25 et 55 min après le pneumopéritoine.

L'étude de Jee *et al*. [22] a comparé deux groupes chacun composé de trente-deux patients subissant une cholécystectomie laparoscopique. Le groupe témoin a reçu de la solution saline et un groupe de magnésium a reçu 50mg/kg de sulfate de magnésium immédiatement avant le pneumopéritoine. Les résultats de cette étude montrent que les pressions artérielles systoliques et diastoliques étaient significativement plus élevées dans le groupe control que dans le groupe de patients recevant le magnésium à 10, 20, et 30 minutes après le pneumopéritoine. Le taux de norépinephrine et de vasopressine moyenne étaient plus élevés dans le groupe control que dans le groupe de magnésium à cinq, et 10 min après le pneumopéritoine. Il n´y avait pas de différence significative entre les groupes par rapport aux taux de rénine plasmatique et de cortisol. Le sulfate de magnésium a montré une efficacité meilleure par rapport à d´autres produits diminuant les perturbations hémodynamiques liés au pneumopéritoine tel que le clonidine. En effet, l´étude de Kamble *et al*. [23] a inclus quatre-vingt-dix patients âgés de 18 à 60 ans, de classification ASA 1 ou 2, proposés pour une cholécystectomie laparoscopique. Les patients ont été repartis aléatoirement en 3 groupes de 30 patients chacun: groupe MG (ayant reçu 50 mg/kg de sulfate de magnésium par injection dilué dans 10 ml de solution saline normale pendant 10 min avant le pneumopéritoine): groupe C (ayant reçu une injection de clonidine de 1 µg/kg dilué dans 10 ml de solution saline normale pendant 10 min, avant le pneumopéritoine) et groupe NS (ayant reçu 10 ml de solution saline normale par voie intraveineuse, pendant 10 min avant le pneumopéritoine). Cette étude a démontré que le magnésium et la clonidine ont tous les deux atténué la réponse hémodynamique au pneumopéritoine. Cependant, le magnésium a atténué mieux la réponse hémodynamique que la clonidine.

Notre étude n'a pas noté des effets indésirables liés à l'administration du sulfate de magnésium. L'étude de Oh *et al*. [24] incluant 714 patients a étudié l´association entre la perfusion peropératoire de sulfate de magnésium et l´incidence d´insuffisance rénale aigue (IRA) après une chirurgie abdominale laparoscopique majeure. Les patients inclus dans cette analyse ont été randomisés en deux groupes équivalents: le groupe MG ayant reçu une perfusion peropératoire de 50 mg/kg de sulfate de magnésium à l´induction suivi par une perfusion de 15 mg/kg/h et le groupe placebo ayant reçu la même quantité en solution saline. Les résultats ont montré que la perfusion de magnésium était associée à une diminution significative de l´IRA postopératoire (intervalle de confiance à 95 %, 0,14-0,94; P = 0,037).

Notre étude a permis la mise en évidence d´une nouvelle utilisation de sulfate de magnésium en tant que vasodilatateur assurant une stabilité hémodynamique au cours de la chirurgie laparoscopique. Il s´agit d´une alternative simple à réaliser et qui n´a pas montré de complications péri-opératoires. Cependant notre étude a présenté quelques limites. En effet, nous n´avons pas dosé les catécholamines et la vasopressine, et nous n´avons pas effectué de surveillance hémodynamique invasive vue qu´ils ne sont pas de pratique courante dans notre établissement.

## Conclusion

Le sulfate de magnésium semble être une technique efficace pour prévenir les perturbations hémodynamiques causées par le pneumopéritoine au cours de la chirurgie laparoscopique.

### Etat des connaissances sur le sujet

- *La création de pneumopéritoine augmente la pression artérielle et la fréquence cardiaque;*
- *Le magnésium bloque la libération de catécholamines de la glande surrénale et des terminaisons du nerf adrénergique;*
- *Le magnésium produit une vasodilatation en agissant directement sur les vaisseaux sanguins; le magnésium atténue la sécrétion de vasopressine stimulée par la vasoconstriction*.

### Contribution de notre étude à la connaissance

- *Le sulfate de magnésium a assuré une meilleure stabilité hémodynamique au cours de la coeliochirurgie;*
- *Le sulfate de magnésium a atténué les variations de la pression artérielle systolique après l´insufflation du pneumopéritoine;*
- *Le sulfate de magnésium a atténué les variations de la fréquence cardiaque après l´insufflation du pneumopéritoine*.

## References

[1] Vecchio R, MacFayden BV, Palazzo F (2000). History of laparoscopic surgery. Panminerva Med.

[2] Blobner M, Felber AR, Gögler S, Feussner H, Weigl EM, Jelen G (1993). The resorption of carbon dioxide from the pneumoperitoneum in laparoscopic cholecystectomy. Anaesthesist.

[3] Hodgson C, McClelland RM, Newton JR (1970). Some effects of the peritoneal insufflation of carbon dioxide at laparoscopy. Anaesthesia.

[4] Richardson JD, Trinkle JK (1976). Hemodynamic and respiratory alterations with increased intra-abdominal pressure. J Surg Res.

[5] Myre K, Rostrup M, Buanes T, Stokland O (1998). Plasma catecholamines and haemodynamic changes during pneumoperitoneum. Acta Anaesthesiol Scand.

[6] Walder AD, Aitkenhead AR (1997). Role of vasopressin in the haemodynamic response to laparoscopic cholecystectomy. Br J Anaesth.

[7] McLaughlin JG, Scheeres DE, Dean RJ, Bonnell BW (1995). The adverse hemodynamic effects of laparoscopic cholecystectomy. Surg Endosc.

[8] Lishajko F (1970). Releasing effect of calcium and phosphate on catecholamines, ATP, and protein from chromaffin cell granules. Acta Physiol Scand.

[9] Altura BM, Altura BT (1978). Magnesium and vascular tone and reactivity. Blood Vessels.

[10] Laurant P, Touyz RM, Schiffrin EL (1997). Effect of magnesium on vascular tone and reactivity in pressurized mesenteric resistance arteries from spontaneously hypertensive rats. Can J Physiol Pharmacol.

[11] Takata M, Wise RA, Robotham JL (1990). Effects of abdominal pressure on venous return: abdominal vascular zone conditions. J Appl Physiol (1985).

[12] Antoniou EA, Kairi E, Margonis GA, Andreatos N, Sasaki K, Damaskos C (2018). Effect of Increased Intra-abdominal Pressure on Liver Histology and Hemodynamics: An Experimental Study. In Vivo.

[13] Banerjee A, Saini S, Lal J (2021). Evaluation of hemodynamic changes during laparoscopic cholecystectomy by transthoracic echocardiography. J Anaesthesiol Clin Pharmacol.

[14] Goodale RL, Beebe DS, McNevin MP, Boyle M, Letourneau JG, Abrams JH (1993). Hemodynamic, respiratory, and metabolic effects of laparoscopic cholecystectomy. Am J Surg.

[15] Gannedahl P, Odeberg S, Brodin LA, Sollevi A (1996). Effects of posture and pneumoperitoneum during anaesthesia on the indices of left ventricular filling. Acta Anaesthesiol Scand.

[16] Vickovic S, Pjevic M, Uvelin A, Pap D, Nikolic D, Lalic I (2016). Magnesium Sulfate as an Adjuvant to Anesthesia in Patients with Arterial Hypertension. Acta Clin Croat.

[17] de Baaij JHF, Hoenderop JGJ, Bindels RJM (2015). Magnesium in man: implications for health and disease. Physiol Rev.

[18] Houston M (2011). The role of magnesium in hypertension and cardiovascular disease. J Clin Hypertens (Greenwich).

[19] Yamori Y, Taguchi T, Mori H, Mori M (2010). Low cardiovascular risks in the middle aged males and females excreting greater 24-hour urinary taurine and magnesium in 41 WHO-CARDIAC study populations in the world. J Biomed Sci.

[20] McCarty MF (1996). Complementary vascular-protective actions of magnesium and taurine: a rationale for magnesium taurate. Med Hypotheses.

[21] Showket AD, Das Gupta D, Deopujari RC, Gomes P (2022). Effect of Magnesium Sulphate on Attenuation of Hemodynamic Stress Responses during Laparoscopic Abdominal Surgeries. Cité 31 mai.

[22] Jee D, Lee D, Yun S, Lee C (2009). Magnesium sulphate attenuates arterial pressure increase during laparoscopic cholecystectomy. Br J Anaesth.

[23] Kamble SP, Bevinaguddaiah Y, Nagaraja DC, Pujar VS, Anandaswamy TC (2017). Effect of Magnesium Sulfate and Clonidine in Attenuating Hemodynamic Response to Pneumoperitoneum in Laparoscopic Cholecystectomy. Anesth Essays Res.

[24] Oh TK, Oh AY, Ryu JH, Koo BW, Lee YJ, Do SH (2019). Retrospective analysis of the association between intraoperative magnesium sulfate infusion and postoperative acute kidney injury after major laparoscopic abdominal surgery. Sci Rep.

